# English‐Only Policies and Allegations of Racism in Nursing: Safety, Culture and Respect Prevail

**DOI:** 10.1111/jan.16813

**Published:** 2025-02-12

**Authors:** Sharon Brownie, Linda Chalmers

**Affiliations:** ^1^ Swinburne University of Technology Melbourne Australia; ^2^ Waikato Institute of Technology (Wintec) Hamilton New Zealand; ^3^ Ngāti Rangitihi, Ngāti Pū, Ngāi Te Rang Bay of Plenty and Hauraki New Zealand; ^4^ Independent Researcher Auckland New Zealand

**Keywords:** acculturation, communication, English‐Only policy, human rights, migration, nursing, patient safety, professionalism

## Abstract

**Aims:**

To provide a critical analysis of the allegation that introduction of workplace English‐Only policies for nurses may be racist. To provide guidance to inform policy development in this field.

**Methods:**

The intertwined complexities informing English‐Only policy development are explored inclusive of the complicated relationship between patient safety, human rights, cultural context, ethics and the social norms which guide manners and respect.

**Results:**

Communication failures are confirmed as a major cause of patient harm incidences with adverse events extensively exacerbated in the absence of shared language between nurses, patients, families and the broader healthcare team. The combined global movement of nurses and increasing diversity within clinical teams points to heightened risk of communication failure.

**Conclusion:**

Unequivocally, patient safety (inclusive of cultural safety) confirms the necessity for policies related to shared language in health. Safety‐orientated English‐Only policies are neither racist nor a breach of human rights. Whether it be in the skies, on the sea, or in a healthcare context, shared language policies and standards (most commonly English‐Only policies) are needed to protect human safety and avoid harm.

**Implications for the Profession and/or Patient Care:**

The demographic profile of most western and many developing nations has changed significantly over the last two decades adding new complexity in healthcare contexts. Need exists for shared language policy in nursing with priority to safety, human rights, respect, ethical recruitment and to the social and cultural dimensions of the workforce.

**Summary**

- This discursive seeks to contribute to discussion about the need for English‐Only or other shared language policies in health.
- Shared language policies and standards are protective in mitigating the risk of communication related adverse incidents and harm.
- Findings contribute to quality and safety in care and are of relevance to nursing and health service management leaders nationally and globally.

## 1 Introduction

In October 2024, a media storm erupted in New Zealand following issue of workplace English‐Only policy directives from a chief nurse in one of the nation's largest health districts, followed by reports of similar English‐Only directives to staff at two other health districts. One news headline asked is it ‘Culturally intolerable’ for nurses not to speak English? or ‘racist’ to insist? (Kavanaugh 2024). Reference to a sample of the news articles illustrates the intensity of argument which broke out following endeavours to stop nurses using languages other than English in the clinical setting. In reviewing the news items it can be noted that some health leaders defended the directives based on the rationale of ensuring clinical quality and patient safety (Akoorie 2024; Davison and Gabel 2024; Gabel 2024; Health Reporter RNZ 2024; Martin 2024). In contrast, the articles profiled a range of public opinion and experiences of nurses and families annoyed or distressed by the behaviour with counter claim accusations of racism and bullying of foreign nurses. The reporting also drew political opinion, some conciliatory toward internationally qualified nurses (IQNs) using their native language in practice and others vague as to the professional and clinical implications of doing so.

Issues and challenges associated with consistent and competent use of a shared medical language and clinical terminology in nursing practice are not new. The need for a commonly shared language (often referred to as either ‘clinical’ or ‘medical’ language) among nurses, patients, families and other health professionals is known to be critical to delivery of high quality and safe healthcare (Hull 2016). The increasing focus on the need for a universally understood language is undoubtedly linked to the high international mobility of registered nurses, particularly in nations with an over reliance on IQNs. The issue is intensified with the extensive movement of nurses from low and medium income countries to high income countries (Viken et al. 2018).

What may be seen as a straightforward policy matter is in fact highly complex, requiring comprehensive analysis and discussion in the context of large‐scale global migration and increasing multilingual health service contexts (Pun 2023). In this discursive, we respond to the allegation the English‐Only policies for nursing contexts are racist by examining a selection of the issues and challenges associated with increased diversity among the nursing workforce, in particular IQNs for whom English is a second language (hereafter we refer to this nursing workforce group as ESL‐IQN). We draw perspectives and evidence from a wide range of peer‐reviewed literature, media, policy and legislation spanning several decades and demonstrating that strengthened policy for shared language use in healthcare contexts, including New Zealand, are urgently needed. As diverse and growing global populations will invariably require more diverse and growing nursing workforces, we also advance some broad recommendations for enhancing supportive professional acculturation of ESL‐IQNs in New Zealand and elsewhere.

## 2 IQNs in New Zealand's Unique Cultual and Language Context

New Zealand has the highest reliance on IQNs in the western world. The World Health Organization's first ‘State of the World's Nursing’ report (World Health Organization 2020a) drew data from 191 member states and showed New Zealand's total nursing workforce was 27.25% ‘foreign trained’. This showed New Zealand as having the highest percentage of IQNs of countries among the countries surveyed (World Health Organization 2020b). Since this time, and based on data from the Nursing Council of New Zealand (NCNZ), registration and recruitment of IQNs has ballooned, with 46.3% of registered nurses with practicing certificates being internationally qualified by September 2024 (NZNC 2024c). Although the NCNZ also advises that some of these nurses, who have practicing certificates, may not, or not yet be, practicing in New Zealand.

English is the predominant de facto official language used in New Zealand as a corollary of British colonisation of the country in the 19th century (Benton 2023). Te reo Māori (the Māori language) the language of the indigenous Māori population is also an official language via the Māori Language Act 1987 and 2016 (New Zealand Government, 1987; 2016). Establishment of te reo Māori as an official language in New Zealand's domestic law, also reflects fundamental obligations on New Zealand Governments to uphold responsibilities under the Treaty of Waitangi to protect Māori culture, within which Māori language is an integral part (Waitangi Tribunal 1986). New Zealand Sign Language became the third official language by virtue of the New Zealand Sign Language Act 2006 (New Zealand Government, 2006) and with this elevating the rights and needs of hearing impaired people in New Zealand society. Although English, Māori and New Zealand sign language are the de facto and official languages of the country, multiple other languages are active in communities, including those of several Pacific Island and Asian nations following substantial migration and settlement of peoples from these regions.

New Zealand has an increasingly diverse population in terms of ethnicity. The 2023 Census, while recording European to be the largest ethnic grouping (67.8%), also identified that there were growing numbers of Māori, Asian, Pacific peoples and Middle Eastern/Latin American/African (MELAA) ethnicities, each of which grew significantly faster than the European ethnic group. Those reporting Māori ethnicity accounted for 17.8% and one or more Asian ethnic groups 17.3% of the total population (Stats New Zealand 2024). Migration‐driven increases in the Asian population are particularly apparent, with an additional 153,973 people reporting Asian ethnicity in 2023 compared with the 2018 Census (RNZ 2024). In addition, growing numbers of people report more than one ethnicity.

Although all three official/de facto official languages are used in health services in New Zealand, English is the formally shared or common language of the health sector. Healthcare professionals who train in New Zealand are taught and practice their clinical vocation in the English language. ‘Medical language’, is a universal construct in healthcare utilised by multidisciplinary health professionals requiring service context and discipline specific proficiency that conveys or interprets complex technical information and deeper meanings than lay language (Hull 2016). The common or shared medical language of healthcare in New Zealand is English. Finch (2014) specifically frames this as ‘medical English’ even in a nursing practice context. Where a shared language for healthcare is not in use in multilingual, multidisciplinary healthcare teams, patient and healthcare team language disconcordance and team disengagement may occur resulting in language based communication barriers and failures along with clinical errors (Hull 2016).

In the unique context of New Zealand, aspects of the Māori language are taught or introduced throughout health professional training and may be utilised in conjunction with English in models of care and delivery. Section 9 of the Māori Language Act provides ‘that “as far as is reasonably practical” the language should be used in the promotion of government services and provision of information to the public (9.1.b) and that “government services and information should be made accessible to iwi and Māori through the use of appropriate means (including the use of the Māori language)” (9.1.c)’ (Benton 2023, 578). The emphasis on utilising te reo Māori in healthcare in New Zealand is to enhance culturally safe and congruent care for Māori patients and their families. Incorporating Māori language and models of care in New Zealand's health system is also underpinned by the necessity to reduce substantial and enduring health inequities experienced by Māori (Minister of Health 2023) and reflected in the core health system legislation for New Zealand in the Pae Ora (Health Futures) Act (2022); New Zealand Government, (2022). The NZNC most recent standards for nursing education programmes include the requirement for education providers to integrate Māori models of health, language and culture in their curriculum (NZNC 2024d).

As with many other healthcare systems internationally, in circumstances where a patient does not communicate in English, healthcare staff are largely reliant on professional interpreters, or family members with bilingual ability to assist with translation of care delivery or need to patients. Increasing cultural and linguistic diversity in New Zealand's health workforce means that a number of health staff may utilise their multiple or bilingual language abilities to support communication to patients that do not speak, read or comprehend the English language. The NCNZ recognises this diversity stating that ‘… there will be times when it is appropriate to communicate in other languages including Te Reo Māori. We support diversity and inclusiveness in the nursing workforce to meet the needs of our diverse population’ (NZNC 2024b).

Recognising that clinical, cultural and emotional safety is paramount, the Health Practitioners Competency Assurance Act 2003 (New Zealand Government 2003) requires the NCNZ to be satisfied that a nurse can communicate at ‘an appropriate level to practice in their scope … and that their ability to communicate and comprehend English is sufficient to protect the health and safety of the public’ (NZNC 2022a) p1. Nurses from the UK, USA, Canada, Ireland and Singapore meet English language competency standards for registration in New Zealand through a nursing education evidence pathway, or nursing registration evidence pathway (although some nurses who originate from these jurisdictions may also complete English language testing) (NCNZ 2022). All other IQNs need to complete an evidence based testing pathway, via either the International English Language Testing System (IELTS) or Occupational English Testing System (OETS). Thus, IQNs registering by way of an evidence based testing pathway, must have met minimum standards of competence in reading, comprehending, speaking and writing in English.

Although establishing and enforcing standards of English language competence by the NCNZ is reassuring, it does not necessarily mean that ESL‐IQNs consistently use English in their everyday clinical practice with patients, families or health professional colleagues. A range of issues are associated with the extensive growth of ESL‐IQNs in New Zealand's nursing workforce and healthcare system. As the media reporting from New Zealand has highlighted, some ESL‐IQNs are communicating with each other in their native languages in clinical settings where patients, family and other staff may hear and observe, but not understand and may feel excluded or offended. It is vital to understand why this may be occurring, the possible impacts on quality and safety of care as well as the social and cultural environment, and how it should be addressed.

## 3 Nursing Perspectives on Use of Non‐English Mother Tongues in the Workplace

Authors noted limited information on this topic within peer‐revised literature. However, similar to New Zealand, discourse is occurring internationally via media, in particular social media where debate and opinion among nurses and other healthcare professions is common. A range of social media‐based conversations was identified by the authors about nurses communicating in their mother tongue from contributors in the UK and North America over the last 10 years. Discourse is particularly prevalent on the Reddit platform with participants articulating a range of views using non‐descript online identifying names. Examples (summarised in Table 1) include entry titles such as ‘English is the only language acceptable’ (Partyhardypillow 2021) or ‘Feeling excluded by my multilingual nurse buddies’ (StrongTXWoman 2024). In another, ‘Nurses talking in their own language’ (ichbinmatt 2016) a nursing student describes feeling socially isolated by registered nurses who speak their own different language but not wanting to be the one to complain about it. Other commentary raises the question ‘Is it reasonable to fire someone for speaking another language in the workplace’ (DribbleKing97 2024). A range of opinion from medical clinicians was sparked in response to a post by an international medical student who was instructed by a nurse to speak English in the wards (KingKong433 2023).

**TABLE 1:** Sample of social media blogs and news comments.

News reporter or blogger	Forum	Headline
DribbleKing97 (2024)	Reddit	Is it reasonable to fire someone for speaking in another language at the workplace
ichbinmatt (2016)	Reddit	Nurses talking in their own language
StrongTXWoman (2024)	Reddit	Feeling excluded by my multilingual nurse buddies
Partyhardypillow (2021)	Reddit	English is the only language acceptable
(KingKong433 2023)	Reddit	Language usage on the ward
Allen (2009)	Las Vegas Sun	Foreign nurses can slip into communication gap
Fleming (2022)	The Guardian	There were moments I questioned my passion for the job: the overseas nurses helping to keep the NHS running
de Loryn (2020)	James Cook University News	International nurses struggling to fit in
Akoorie (2024)	Radio NZ	Fury over Waikato Hospital English‐Only directive to nurses: ‘It's against human rights’
Health Reporter RNZ (2024)	Radio NZ	Language directive a ‘lurch back in time’—health professor
Pennington (2024)	Radio NZ	Indian nurses at Palmerston North Hospital told to stop using native language
Reporter Indian Newslink (2024)	Indian Newslink	Nurses conversing in foreign languages raise concerns

These media insights highlight that use of mother tongues by ESL‐IQNs in front of patients, families, colleagues and team members who do not understand the language being spoken is considered disrespectful, unfair, unsafe and probably against organisational policy. Speaking another language in front of others who do not understand is unsettling and can be considered to be as rude as whispering. In their extensive analysis of regulating foreign languages and monolingualism in the workplace, Teboul and Speicher (2007) identify that these language tensions may include that colleagues and clients alike may think they are being talked or gossiped about or that some vital information is being concealed from them. This perception or experience may undoubtedly be considered disrespectful behaviour, which could be deeply unsettling for patients, families and colleagues (Grissinger 2017).

The demographic profile of most western and many developing nations has changed significantly over the last two decades adding new complexity in healthcare contexts. These changes are significant for nurses for whom understanding cultural diversity and working in multilinguistic contexts is now a daily reality (Campellville University 2016). For most nurses, practice now involves working with many languages and many cultures within a single hospital or healthcare setting (de Loryn 2020). A glance back over the small sample of social media blogs and news headlines show that the issues are not new, however, concerns appear to be increasing as migration increases and diversity continues to grow. Of concern, attempts to address issues in New Zealand have resulted in raised tensions and allegations of systematic racism (Akoorie 2024; Health Reporter RNZ 2024). In the heightened context, the issues are many and complex and something everyone seems to have an opinion including ‘being disrespectful and exluded’ to ‘emboldening racists’ (Reporter Indian Newslink 2024). Clearly there is a need for calm and thoughtful analysis of the priority issues, followed by well‐informed policy development and appropriately aligned support.

## 4 Interwoven Consideration and Complexities

### 4.1 More Than an International English Language Score—Dialects, Accents and Colloquialisms

Although rigorous English language testing is part of the migration and professional registration journey for ESL‐IQNs, even when English is spoken by both the patient and nurse, communication issues can still arise (Allen 2009) due to differing accents, local idioms, vernacular and slang. Opinion about heavy accents and idioms as a barrier to communication with ESL‐IQNs were also noted in media and social media examined above. Often the patients do not understand what they are saying and other nurses may not understand them either (Allen 2009; de Loryn 2020).

The complex challenges of professional practice in a country and culture other than one's own are only experienced after migration, at which time ESL‐IQNs may encounter significant cultural and linguistic hurdles, discrimination and limited career mobility (Kamau et al. 2022; Pressley et al. 2022). Differing turns of phrase, colloquialisms and medical terminology (Pressley et al. 2022) contribute to a professional environment that can be isolating and stressful for migrant nurses. Language challenges faced by ESL‐IQNs are well documented in the literature, inclusive of issues associated with English dialect and fluency (Mantley 2021; Montayre et al. 2018; Wells 2013). Where nurses do speak the shared language of healthcare systems or organisations, accent discrimination may also occur (Teboul and Speicher 2007; Teboul 2002). Accent discrimination can occur even when an ESL‐IQN has mastered use of English to the required level for professional practice, but where their accent may trigger perceptions of low intelligence, unsuitability to lead and downgrading by employers (Iheduru‐Anderson 2020). In addition to being stereotyped, this may result in loss of confidence and self‐esteem (Uzun 2023).

Research suggests that international nurses long for exchanges in their first language to combat disempowerment and isolation and articulate their identity (Kishi et al. 2014; Philip et al. 2019). This need for a sense of familiarity and belonging may help to explain any propensity for ESL‐IQNs to converse with each other in their mother tongue in clinical settings. Other scholars in management and social sciences have also noted this propensity (Speicher 2002; Teboul and Yoon 2019; Teboul and Speicher 2007). Three insights from this literature include the following: it may be an unnatural experience not to speak in one's native language with others from your culture; some bilingual speakers may unconsciously switch to their original language with members of their own cultural group; and bilingual people may continue to think and converse internally in their native language even when queried in a different language. Further, some ESL‐IQNs make speak a native language where there are multiple dialects (Pun et al. 2017), and this may further complicate nursing communication. A context in which multiple languages are used also increases the risk of error, particularly where nurses are not consistently and competently using English in their nursing practice.

### 4.2 Nurse‐to‐Nurse Communication

Various types of nursing team communications are distinguishable in the literature including formal communication which occurs between nurses on the same shift and across different shifts; communication between nurses on different wards or care units, for example, from an emergency department to theatre reception when patients are transferred for surgery; and informal communication such as when nurses take a meal break. A major component of nursing communication occurs during ‘nursing handover’ or ‘hand off’ (Friesen et al. 2008; Galatzan and Carrington 2018). Communication risks at the time of handover include the risk of information loss, miscommunication and information inaccuracy. Using a language verbally or in written form that is not shared by all nursing team members providing, coordinating or monitoring care delivery to the patient is unsafe practice and heightens risk of communication error or failure. Nursing handover involves transfer of responsibility and accountability (Friesen et al. 2008) between nurses and other clinicians who are professionally responsible and accountable for the clinical information that they give and receive. It is critical that shared language common to all nurses is used in these contexts.

Such is the importance of high‐quality nursing handover that various nursing tools have been developed, extensively tested and evaluated by the profession. These may be utilised to standardise the process to eliminate risks and errors in nursing communication and with this reduction in patient harm (Galatzan and Carrington 2018; Goldsmith et al. 2010). The Australian Commission on Safety and Quality in Health Care (n.d.) established standards for structured clinical handover including the various types of clinical handover and the recommended content reinforcing the importance of the quality of handover practice (Australian Commission on Safety and Quality in Health Care n.d.).

Language disconcordance in multilingual healthcare teams contributes to language barriers and patient safety risks (Hull 2016). The concept of language concordance and disconcordance in healthcare literature and research is dominated by study of the challenges of safe care delivery for patients who are the ethnic minority and speak minority languages, particularly Spanish or French in the United States and Canada (de Moissac and Bowen 2019; Lor and Martinez 2020). Nonetheless, within the substantial body of research examining language concordance between provider and patient in healthcare, the evidence powerfully links language concordance to better patient outcomes and experience (Lu 2018; Wu et al. 2021).

Although there are studies that perceive quality and safety concerns associated with ESL‐IQNs, these are based on reported perceptions of patient safety and harm, in contrast to measurement of actual harm (Lor and Martinez 2020). This gap may be due to both methodological as well as ethical challenges within nursing, quality and health research communities. Data associated with patient harm that has second or foreign language communication failure or error as a causative or contributing factor would sit at deep levels of healthcare system incident and complaint management and reporting systems, rendering it less amendable, less appropriate and more contentious to research. Further, it may be virtually impossible to determine where ESL‐IQNs or other professionals have erred in their clinical communication when an error occurs in a language other than English. Clinical incident and complaint management systems in New Zealand use English, offering another compelling reason to utilise a common shared medical language (other than for patients who require translation to their non‐English language) in clinical practice.

### 4.3 Communication Across the Healthcare Team

Some of the criticism that has been identified in the media analysis includes ESL‐IQNs who use their native language in wider nursing and healthcare team situations. Diversity is not confined to nursing teams alone, for example, New Zealand's medical workforce is also diverse with 40% having qualified overseas and originating from more than 100 countries, many of whom also speak English as a second language (Medical Council of NZ 2024). Nurses may also use their native language to communicate regarding patients and their care with these team members who share the same or differing native tongue. Wider and important members of the healthcare team include allied health, technical, administrative and managerial staff, many of whom may also have English as their second language. Shared language responsibilities for communicating in the totality of a multidisciplinary team environment remains.

### 4.4 Nursing Communication With Patients and Families

Increasing migration brings with it increasing population diversity including patients who are English second language speakers and who may have limitations to their English language proficiency (Twersky et al. 2024; Ortega et al. 2022). This necessitates translation services, a need for increased workforce diversity and staff who have the competence to speak in a non‐English native languages representative of changing patient populations. In a growing number of jurisdictions, language diversity in nurses is actively sought after with the ability of a nurse to connect with patients and families in their mother tongue highly valued (Kluwer 2024; Zaman 2013). It is important that nurse leaders and policy makers understand the value and importance of workforce diversity and the need for a workforce that mirrors the community being served (Stamps 2024).

The bedside handover of patients is a vital opportunity and point of safety to ensure that the patient and their family are enabled to hear about their status, to understand what is occurring or planned, to contribute to clinical care. It is also a checkpoint to identify when nurses maybe sharing inaccurate information (Australian Commission on Safety and Quality in Health Care n.d.; Friesen et al. 2008). Hand over between nurses at the patient's bedside, in a language that patients and families do not understand has real potential to be clinically unsafe, and to deny patients and families of these important communication opportunities.

Vulnerable patients, for example, those in pain, those with cognitive or physical impairments, and patients who are isolated (e.g., for infection control reasons), are also vulnerable to the impact of provider and patient power asymmetry (Hemberg and Sved 2021). This power asymmetry can be exacerbated when nurses who can speak the patients language, speak over or in front of them in languages that they do not understand. The need for caring, therapeutic and sensitive communication and the establishment of respectful relationships between healthcare provider and patient are crucial for all clinicians where English is a second language (Finch 2014). Language is an expression and manifestation of culture, including healthcare culture where patients and families must have trust and confidence in those who care for them, including by way of communication in a shared language between patient and healthcare provider (Hemberg and Sved 2021).

### 4.5 Cultural Safety

In the New Zealand context, registered nurses are obligated to practice in a manner that is culturally safe, with this being a specific requirement of competence to practice (NZNC 2024a). Cultural safety is defined as:

‘The effective nursing practice of a person or family from another culture, and is determined by that person or family. Culture includes, but is not restricted to age or generation; gender; sexual orientation; occupation and socioeconomic status; ethnic origin or migrant experience; religious or spiritual belief; and disability’ (NZNC 2011).

Cultural safety and kawa whakaruruhau (cultural safety in a Māori context) requires nursing clinicians to reflect on and identify their own culture, attitudes and experience and how that impacts their nursing practice (NZNC 2011, 2024b, 2024d). Cultural safety also requires nurses to resolve cultural tension between themselves and the people served and negotiate power imbalances between themselves and consumers to provide effective, equitable and acceptable service, which ‘… minimizes risk to people who otherwise might be alienated from the service’ (NZNC 2011) p10. Nurses speaking in their native tongue, and with this, effectively excluding patients and their families from the communication, could also be considered culturally unsafe nursing practice, particularly when patients or family members demonstrate or express that the behaviour is distressing or wrong. Culturally safe nursing practice addresses the power differential that clinicians have over patients and their families, and in doing so also uplifts crucial ethical principles and practice in healthcare such as autonomy, beneficence, non‐maleficence, truth telling, justice and self‐determination (Varkey 2021).

Lu et al. (2018) speak to the ‘sociopragmatic’ and ‘pragmalinguistic’ aspects of culturally appropriate communication. Sociopragmatic language aspects relate to the cultural expectations and values inherent within interactions in differing cultural contexts, such as the sociopragmatic norms in respect to what constitute appropriate, mindful manners and professional behaviours^1^. Pragmalinguistic language considerations involve the ability of a speaker to utilise linguistics meanings and nuances within a given language and cultural context. Sociopragmatic and pragmalinguistic aspects of communication are deeply intertwined and both are needed for culturally appropriate communication and care (Lu 2018). Subsequently, ESL‐IQNs require time and acculturation support to achieve cultural safety competence specific to their new practice context (Brunton et al. 2019; Chun 2019; Pressley et al. 2022; Thirlwall et al. 2021).

### 4.6 Professional Respect

The use of shared language is a matter broader than safety alone. Arguably, one of the most important criticisms arising from assessment of the media discourse of ESL‐IQNs speaking in their native tongue in clinical practice is when this occurs in front of patients, families and colleagues who have no understanding of what is being said. Quotes highlighted in Table 2 are illustrative of the exclusion and distress experienced by patients and families when this occurs.

**TABLE 2:** Patient and family reactions to foreign languages.

*Liz said that repeatedly, foreign nurses and caregivers spoke in their own language to each other, in her presence while with her mother was dying. ‘It was unsettling and divisive. I could have well done without that’, she said*. *Another person wrote, saying, ‘Medical staff should only speak English, Te Reo or Sign Language. I have experienced Asian nurses having conversations in their own language. I felt threatened as I did not know what they were saying. It is so unprofessional of them. If they choose to come to New Zealand, they must speak English!’* *One person who had been in and out of Wellington Hospital over the past year said that they had complained when staff once spoke their own language in front of them*. *‘I felt disrespected and excluded. I complained and received an apology. I am supportive of nurses or hospital staff speaking their own language when away from patients, off the ward, or staff room’, the person said*.

*Source:* Reporter Indian Newslink (2024).

Professional colleagues can feel equally excluded when languages other than their own is spoken in ward settings, team rooms and lunchrooms. Loss of the sense of belonging is an emerging phenomena when the balance between local and inwardly migrating populations change and local populations become minority groups (Brownie et al. 2024).

### 4.7 Social Exchange in the Workplace

Other aspects of the care team environment include non‐clinical situations, for example in lunch rooms or cafeteria. These social exchanges (Taboul and Yoon 2018) often occur in languages other than English involving small talk and chit chat that is not work related. Commentary regarding this was also evident in social media. In this environment nurses and other staff use of native tongues can be very beneficial to the well‐being of internationally qualified healthcare staff affirming social identify and belonging. This is a significant but not new issue as it may also be viewed negatively and work against cohesion and trust in healthcare teams fostering divisiveness and suspicion and loss of sense of belonging (Fink et al. 1996). Culturally competent nurses are attuned to sociopragmatic aspects of language use within the culture in which they are practicing (Lu 2018). Culturally competent nurses understand and respect local norms knowing that it is considered exclusionary and rude to speak in a language to the exclusion of other language speakers.

### 4.8 Workplace Balance and the Sense of Belonging

Just over 46% of New Zealand's nursing workforce are now IQNs and nursing teams may be comprised of equal or even greater numbers of IQNs, including ESL‐IQN, than their domestically trained counterparts. In this context, ESL‐IQNs may form the majority. There is a natural tendency for nursing colleagues from similar cultures to cluster together. This occurs across both migrant groups and within New Zealand domestic nursing cohorts, some of whom will also be from culturally and linguistically diverse backgrounds such as Māori and Pacific Island nurses. However, when clusters form, there is a risk of cultural ‘otherness’ wherein a dominant group (us) relates to out‐groups as (them or others) with potential to stigmatise the difference with negative impact on the sense of belonging (Roberts and Schiavenato 2017). Use of language which is not understood by the full team increases the risk of ‘otherness’. The need for and experience of belonging, and a sense of community in nursing (Patel et al. 2024), is associated with improved job satisfaction, less burnout and job stress (Ditzel 2017). Protecting the sense of belonging for any cultural subgroup in nursing requires sensitive and strategic attention to ensure that there is balance of safety for staff, for patients and their families, as well as cohesiveness and nurturing in professional nursing practice.

### 4.9 Human Rights

Human and employment rights obligations add to challenges and responsibilities associated with culturally and linguistically diverse workplaces and workforces. Debate, litigation and scholarship of these rights is well advanced in both North America and the UK, although not necessarily in a healthcare context (Higgins 2003; Speicher 2002). Prohibition on use of the native language for ESL‐IQNs, may result in a sense of discrimination without clear justification. This assertion is undoubtedly made within the New Zealand media commentary but without the careful analysis and clarity essential to understanding and addressing the phenomena.

Key human rights instruments applicable to language use of ethnic minorities in New Zealand, include the New Zealand Bill of Rights Act, section 20 Rights of Minorities (New Zealand Government 1990), and the International Covenant on Civil and Political Rights article 27 (Benton 2023). Importantly, however, this is a right to communicate in a minority language (e.g., in Arabic, Samoan, Hindi, Afrikaans, Swahili or Tagalog), rather than a language right per se (Benton 2023).

The New Zealand Human Rights Act (1993); New Zealand Government (1993) makes it unlawful for employers to treat employees unfairly because of ethnicity or first language use. The Human Rights Commission describes that trying to stop an employee from speaking their first language at work, may be discrimination and that there are limited situations where an English‐Only policy is justified (Human Rights Commission n.d.). The overlay of human rights and language use in association with employment in New Zealand therefore, is highly complex, and requires healthcare organisations in New Zealand to be well informed of their legal obligations. In short, introducing English‐Only policy in nursing or other healthcare professions must be done with clear justification, with balance and attention to the social and cultural dimensions of the workplace, the workforce and the patients and families who are served.

Communicating in a language the patient does not understand is also a potential failure to meet the patient's rights and expectations enshrined in the Code of Health and Disability Services Consumer Rights (Health and Disability Commissioner 2022). At least four consumer rights in the code are relevant to this analysis: the right to treated with respect; the right to services of an appropriate standard; the right to effective communication; and the right to be fully informed.

Significant emphasis in the New Zealand health system is given to ensuring the patient has information about their health status and healthcare plan in a language that they understand and from which they can make informed decisions. Failure to achieve these rights because of language barriers, which should be mitigated by nurses competent to practice in English, could be considered a breach of the patient's rights. It is one of the many rationale for the use of interpreters where patients or their family do not comprehend English.

However, it may be a breach of human rights to unreasonably restrict use of a mother tongue in the workplace unless there is a compelling reason to do so. Safety is identified as a compelling reason, whereas a speak English‐Only rule applied to staff not involved in work duties or on a break would be unlawful (Human Rights Commission n.d.; US Department of Labour 2024).

## 5 The Safety Imperative

Safety is unarguably identified as the compelling issue related to the need for language policy in health care settings. Clear links exist in respect to use of a shared language and safety in care provision including clinical, cultural and psychosocial safety. Communication failures or errors are noted as the most enduring, prevalent and complex causes of poor quality care and patient harm (Guttman et al. 2021; Burgener 2017; van Rosse et al. 2016; O'Daniel and Rosenstein 2008; Donaldson et al. 2017; Committee on Quality Of Health Care in America 2000); Chalabian and Dunnington 1997). Language barriers between healthcare providers and patients are a significant dimension of communication error and/or failure with a substantial literature found in the last decade dedicated to understanding and addressing the challenges (Finch 2014; Hemberg and Sved 2019; Ortega et al. 2022; van Rosse et al. 2016), much of which has been driven by the increasing global mobility of both populations and health workforces. O'Daniel and Rosenstein (2008) succinctly describe that ‘When health care professionals are not communicating effectively, patient safety is at risk for several reasons: lack of critical information, misinterpretation of information, unclear orders over the telephone, and overlooked changes in status’ (O'Daniel and Rosenstein 2008, 271). Further, when clinicians do not understand what patients or family are saying, they struggle with their role and may feel inadequate in their ability to provide care (Kuzemski et al. 2022). A common language is the glue that keeps a system working and safe, particularly in times of crisis when it is critical that all parties must understand each other. Reference to the aviation and maritime sectors illustrate the link between shared language and safety considerations.

### 5.1 Aviation English: The Language of the Skies

In a bid to improve communication safety in international airspace, the 1944 Convention on International Civil Aviation (Chicago Convention) established core principles regarding English as the common language for all aviation personnel. Drafted by 54 member states, the Convention came into effect 4 April 1947 following ratification by the required number of nations formalising English as the recognised language of the skies (ICAO n.d.). The Convention was strengthened in 1959 at which point the International Civil Aviation Authority (ICAO) published English speaking guidelines inclusive of technical nomenclature related to aviation, formally known as Aviation English. ICAO recommendations were mandated in 2008 with the introduction of compulsory English competency testing for pilots, air traffic controllers, cabin crew and other key airline staff. Compulsory testing followed several major accidents in which communication errors were identified as the primary cause of disaster. Subsequently, the standard of Aviation English is rigorously maintained with specific aviation phraseology closely monitored. Aviation personnel also undergo detailed training to speak more closely and modify accents to reduce the risk of accent related error. ICAO Standards require English speakers to have a dialect or accent which is ‘intelligible to the aeronautical community’ (ICAO n.d.), the key imperative being safety.

### 5.2 Maritime English: The Language of Seafaring

Maritime English also known as Standard Maritime Communication Phrases (SMCP) is a standardised form of English with nomenclature specific to seafarers and maritime navigation and communication personnel. In 1977, the International Maritime Organisation adopted the Standard Marine Navigational Vocabulary (SMNV) replacing this with the SMCP phrases in 2001 (International Maritime Organization 2024). Both SMNV and SMCP have been developed to address the challenges of shipping movement across international waters. Maritime English provides consistent language for shore‐to‐ship (and vice versa), on board and ship‐to‐ship communications. For obvious safety reasons, the International Convention on Standards of Training, Certification and Watchkeeping for Seafarers (STCW), 1978, requires officers in charge navigating ships 500 gross tonnage or above to be proficient in SMCP to hold navigator's licence (International Maritime Organization 2024). As with Aviation English, there is no suggestion that insistence on use of a shared language is racist—the safety imperative prevails by international agreement.

### 5.3 US English‐Only Policy

Necessitated by large‐scale demographic change, the US grappled with the issue of human rights obligations versus English‐Only Rules more than two decades ago (US Department of Labour 2024). These policy makers note that universally English‐Only policies in a workplace is a violation of human rights and that such policies should be limited to special circumstances only. The USA Equality Employment Opportunity Commission (EEOC) Regulation 29 C.F.R. § 1606.7(b) (US Department of Labour 2024), identifies two of these special circumstances as:

- ‘For communications with customers, co‐workers or supervisors who only speak English’.
- ‘In emergencies or other situations in which employees must speak a common language to promote safety’ (US Department of Labour 2024).

### 5.4 Taiwan: English for Specific Purposes (ESP)

Synchronous with global trends, Taiwan has faced a changing population profile and nursing workforce shortages. As population demographics change and an increasing number of IQNs are recruited in Taiwan, the need for an English speaking nursing workforce increased—a need further fuelled by the emphasis of the Taiwanese Government in expanding medical tourism. Even though Taiwan is not an English speaking nation, the need for clinical English specific to health has been strongly recognised with proactive introduction of English for Specific Purposes (ESP) programmes in health (Lu 2018; Wu et al. 2021). Commitment to delivery of these programmes is based on the evidence that when nurses and patients have different language and cultural backgrounds, delivery of culturally appropriate care and communication is a significant challenge which must be proactively addressed across health systems (Lu 2018).

### 5.5 English: The Global Language of Business

English has long been recognised as the language of business for large‐scale multinational companies with language well recognised as one of the most critical issues for global business performance and success (Piekkari et al. 2014). Many multinational companies have mandated use of English in the workplace, for example, Microsoft in Beijing and Japan‐headquartered Nissan. Other English‐only speaking companies include Airbus, Samsung, Technicolour Renault, Honda and many more (Borzykowski 2017; Neeley 2012). An English speaking requirement is also recognised as essential for those wanting to engage in global volunteer networks (Global Volunteers 2021). This is not a throwback to colonialism, rather it is a pragmatic recognition that large businesses and non‐government organisation (NGO) networks are organised globally rather than regionally, and that the multi‐language business systems, record, transactions and communiques simply do not work. Competitive reality demands an ability to interact effectively with a diverse range of customers. Multiple languages can cause a bottle neck if geographically dispersed staff and customers interact but require translation services for day‐to‐day interactions. Common ground of a shared language is needed to avoid miscommunication and reduce business risk (Borzykowski 2017; Global Volunteers 2021; Neeley 2012; Piekkari et al. 2014).

## 6 Policy Recommendations and Implications for Practice

Unarguably, globalisation has markedly changed the demographic profile of most nations with significant change in the ethnic mix of the nursing workforce. The teams in which most nurses now practice are increasingly multinational. Subsequently, there is an urgent need for policies and standards to ensure safety of practice among nurses with varying and multilingual backgrounds.

The need for shared language and sector specific terminology was first acted upon by the aviation sector more than 80 years ago, followed by the maritime sector 40 plus years ago and increasing number of multinational business entities over the past three‐four decades. Almost 10 years ago, authors such as Hull (2016) asserted the need for specification and endorsement of a shared medical language in health (Hull 2016). Taiwan provides an example of a nation which has understood the issues and embraced the need for English for specific purpose in their health sector (Lu 2018; Wu et al. 2021). Each of these examples illustrate safety orientated responses to language challenges for sectors working across borders and in multilingual context. Although late to do so, it is time for the healthcare related entities (including policy, regulatory and provider entities) to work together and show leadership in this field.

Although eligibility for migration and professional registration are dependent upon language competency testing, this does not ensure safe practice. From both a safety and ethical perspective, recruitment should be supported by thoughtfully developed regulation, clear policy expectations, appropriate orientation and incountry acculturation support from nursing systems and healthcare organisations (Brunton et al. 2019; Thirlwall et al. 2021; Viken et al. 2018). Multi‐layered policy development is needed to address the safety, human rights, ethical and cultural issues supporting the need for English‐Only policies to guide health and nursing care provision (see Table 3).

**TABLE 3:** Policy considerations and implementation requirements.

Policy considerations	Implementation requirements
Safety first	Universal agreement in respect to the need for shared language in healthcare Agreement and action for both national and international guidelines on shared and/or English‐Only language in health Standards developed by professional entities and embedded in relevant legislation
Human rights	Standards which clearly articulate the circumstances in which an English‐Only policy may be applied ○ Standards linked directly to quality and safety Clarity regarding the right to speak other languages ○ Voice & supports for patients and families and their right to be communicated with in a way they can understand ○ Link with respect for local social conventions and norms
Ethical recruitment	Active workforce planning to reduce over reliance on IQNs Ethical recruitment of IQNs to ensure maintenance of balance with population demographics Appropriate orientation and acculturation of migrating nurses
Culture & context	Communication and language competency development strengthened across all nursing education programmes Investment in domestic health workforce development Formal IQN acculturation programmes relevant to local culture and norms

### 6.1 Regulation and Policy

Strengthened legislation and regulation is needed regarding shared language(s) in healthcare. In the New Zealand context, the rapid growth of the ESL‐IQN workforce should alert the NCNZ of to the need for policy review and development in this area. Such work may include reconsideration of the Council's recent move to lower the English language written competency requirement from a previous score of 7.0 to 6.5 in the IELTS and from 350 to 300 in the Occupational English Test (OET). Listening score requirements remained unchanged (NZNC 2022b). Monitoring of ongoing language and cultural safety competence for all nurses practising in New Zealand may also be considered appropriate.

In keeping with the notion of a safety first priority, policies in support of establishing a safety culture are of primary importance (AHRC 2023). In addition to the safety imperative, policy guidelines are needed regarding politeness, respect, culturally defined manners and expected etiquette‐based norms. Equally, nationally orientated regulations should be developed to ensure proactive management of the nursing workforce to reduce over reliance on IQNs. Accurate data is critical to health service and workforce planning with New Zealand urgently requiring better planning process (Brownie and Broman 2024; Brownie et al. 2014). Recent policy advice to the New Zealand Minister of Health provides valuable guidance regarding communication and sociocultural safety in the regulated health workforce in New Zealand (Ministry of Health 2024). Actions at this level, will aid policy development to better mitigate the risks associated with New Zealand's substantial over reliance on IQNs and avoid unethical recruitment of IQNs. From a global perspective, opportunity exists for either and/or the International Council of Nurses and World Health Organization to work with member states to develop international conventions, standards and testing criteria in much the same way as the International Civil Aviation Authority worked with the Aviation community or the International Maritime Organisation worked with those responsible for safety on the seas.

### 6.2 Education, IQN Transition and Acculturation

Passing an English language test at the point of migration and registration is insufficient to establish safe practice. A strengths‐based approach recognises that additional orientation, organisational and collegial support maximise transition to new environments (Ministry of Education 2025). These are key factors in determining successful acculturation and retention of ESL‐IQNs. Supportive colleagues are particularly important (Chun 2019; de Loryn 2020). IQN transition realities are challenging involving significant adaptation with experience of ‘expert to novice’ wherein experienced IQNs need to engage in significant orientation and new learning (Deegan and Simkin 2010). Closer attention to acculturation is needed including systems for ongoing English language proficiency. Time is a key factor with literature pointing to the need for a minimum 3–6 month period of acculturation processes including education related to local legislation, context specific standards, culture and local norms (Chun 2019; Philip et al. 2019; Pressley et al. 2022). Further attention to cultural safety training and kawa whakaruruhau for all IQNs should be in place following employment in New Zealand's health system. The now extraordinary multicultural dynamic of New Zealand's nursing workforce and populations makes such an education strategy critical.

### 6.3 Research

Exploration of literature suggests several areas in which there is a current lack of evidence to inform current policy debates related to language use in healthcare settings. For example, the authors were unable to locate empirical work relating to the perspective of domestic nurses' and their experience in the context of large‐scale increases in ESL‐IQNs in the workplace. Many blogs and news pieces with emotive and provocative headlines and allegations are locatable but there is very little research‐based evidence to inform policy and practice. Further, in nations such as New Zealand, where health equity issues challenge indigenous people, research is explicitly needed to identify best practice about how internationally qualified clinicians are or should be orientated to practice safely in the unique cultural, health inequity and indigenous context to which they have migrated^2^.

## 7 Conclusions

In the New Zealand context, well‐intentioned but hasty policy directives created a media storm with unjustified and unhelpful allegations of racism which detract from the real issue of patient safety and respect. Introducing English‐Only policy in nursing or other healthcare professions must be undertaken with clear justification and with balance giving priority to safety, human rights, respect, ethical recruitment and to the social and cultural dimensions of the workforce. Unequivocally, patient safety (inclusive of cultural safety) confirms the necessity for policies related to shared language in health. English‐Only polices informed by imperatives of safety, culture and respect are neither racist nor a breach of human rights. Whether it be in the skies, on the sea or in a healthcare context, shared language standards and policies (most commonly English‐Only policies) are needed to protect human safety and avoid harm. In addition to the unarguable safety imperatives, policy development must include close attention to the characteristics of specific population groups and give voice to the patients and families who are served—respect and good matters also matter.

## Conflicts of Interest

The authors declare no conflicts of interest.

### Peer Review

The peer review history for this article is available at https://www.webofscience.com/api/gateway/wos/peer‐review/10.1111/jan.16813.

## Data Availability

The authors have nothing to report.

## Endnotes

^1^
Manners refer to social norms and rules guiding social behaviour, specifically the way of interacting with others, promoting cooperation, good will and respect. Manners are culturally defined and change over time in tandem with changes in societal values and norms Swarbrick (2013). *Manners and social behaviour*. Te Ara: The Encyclopedia of New Zealand. https://teara.govt.nz/en/manners‐and‐social‐behaviour/print.

^2^
Authors of this manuscript bring several decades of nursing education, practice and research to this discussion. The work is developed via a bi‐cultural partnership with one author of Ngāti Rangitihi, Ngāti Pū, Ngāi Te Rangi descent and the other a 5th generation New Zealander with extensive international experience. Both are New Zealand qualified nurses and PhD researchers with a deep and enduring commitment to the improvement of health equity and the development of health workforce in New Zealand and further research in these fields.
